# I. Amyloid Degeneration of the Kidney.—II. Endo-Cardial and Peri-Cardial Murmurs

**Published:** 1887-07

**Authors:** James Tyson

**Affiliations:** Philadelphia, Pa.


### Southern Medical Record.

A MONTHLY JOURNAL OF PRACTICAL MEDICINE.

All Communications must be addressed to R. C. WORD, M. D., Managing Editor.

"*Quicquid Præcipies Esto Brevis.*"

VOL. XVII. ATLANTA. GA., JULY, 1887. No. 7.

### ORIGINAL AND SELECTED ARTICLES.

### I. AMYLOID DEGENERATION OF THE KIDNEY—II. ENDO-CARDIAL AND PERI-CARDIAL MURMURS.

*A Clinical Lecture.*

BY JAMES TYSON, M. D., PHILADELPHIA, PA.

*Amyloid Degeneration of the Kidney.*

While it is oftentimes difficult to make a diagnosis of any form of Bright's Disease, it is doubly difficult to be sure of the existence of amyloid degeneration. In this case we have found albumen and hyaline casts; but neither of these conditions alone or in combination are sufficient to warrant an opinion as to the variety of the disease present. When we have waxy casts in the urine we can be sure that we have to deal with a chronic disease, but we must look further to make out the type. In this case I make the diagnosis of amyloid disease from the previous history of the patient, for this woman has had well-marked syphilis, which is a very potent cause of the amyloid disease. Chronic suppurative disease of any kind is very likely to cause this form of degeneration, as, for instance, the hip-joint disease of children, of which the amyloid kidney is a very frequent sequel; so, also, the large white kidney is not at all uncommon in tuberculous consumption.

In connection with the discovery of such a cause our diagnosis will be rendered quite sure if we can find evidences of amyloid disease of the liver and spleen, for it is but seldom, or almost never, that the kidneys alone are affected, these two organs usually participating in the degeneration; so that, from the history alone, you can *surmise* but cannot be absolutely sure, unless you find enlargement of the liver and spleen

Ordinarily speaking, splenic dullness is heard, about, under the ninth and tenth ribs, extending about two inches each way. If, then, we find this dull area increased we can rest assured that we have splenic enlargement. In this case the spleen is very much enlarged and it is very sensitive to pressure. The liver dullness commences at the fifth interspace (one interspace higher than normal) and extends an inch and a half below the ribs in the mammary line (an inch and a half lower than normal), while, in the axillary line it extends even lower down; so, that taking the specific history, the concomitant symptoms and the presence of albumen and hyaline casts, I am quite sure that this woman has amyloid degeneration of the liver, kidneys and spleen.

The treatment for this form of degeneration is the treatment of the disease that has caused it. Syphilitic bone disease and tuberculous phthisis are frequent causes, and our therapeutic measures must be directed towards them. If the causative disease has not gone too far—if its ravages can be checked—the amyloid disease can be cured, or at least held in abeyance. It is important to bear this fact in mind, especially with reference to the performance of surgical operations, particularly in the hip-joint disease of children, where early operative interference will have a good effect on the disease. When tubercular consumption is the cause, we can hope for but little, while when due to syphilis, we have a right to expect much from iodide of potassium, which is the drug to be used in preference to mercury. The dose of the potash must be that of a tonic, though a study of each case must be made and the dose regulated by the tolerance of the individual, but patients with amyloid disease will not, as a rule, bear large doses ; but the rule should be to give as large doses as will be borne. This patient has been taking 20 grains, thrice daily, and has improved very much. In making up your mind as to improvement in these cases, it is safer to rely on diminution in the size of the liver and spleen than on the decrease of albumen, which is liable to great variations in quantity without any change in the disease. Rest has a great influence on its excretion, and it is usually found in greater abundance in the evening than in the morning urine ; but the reduction in size of the liver and spleen offer us positive means of forming an opinion on the progress of the case.

Amyloid disease is never idiopathic as may be the other forms of kidney disease ; that is to say, it is never produced by cold, worry, overwork, etc., but must always have a definite cause.

*Endo-cardial and Peri-cardial Murmurs.*

One of the most difficult problems in auscultation is to positively determine, in some cases, whether murmurs that are heard are extra, or intra-pericardial ; it is admittedly difficult in theory, but it is much more difficult in practice. I present this patient to demonstrate this difficulty.

She has not been well foi three weeks ; there is a beating or throbbing in the epigastric region, pain in the shoulder, extending to the hand, and the arm is so sore as to preclude work; in a word, she has had rheumatism, for which she was treated in the house. About the first of January a to-and-fro murmur was detected near the base of the heart, which was thought to be extra-cardial; while most distinct near the base, it was not transmitted into the carotids, as it would have been had it been intro-cardial. A feature of extra-cardial murmurs, as distinguished from intra-cardial, is that they rapidly disappear ; they possess a fleeting character; they may be here one day and gone the next ; but we do not have these peculiarities with endo-cardial murmurs. Towards the apex, in this case, we have no murmur at all. As a rule, pericardial murmurs are less rough than endo-cardial, but this is not invariably so, while fremitus, as conveyed to the hand, is much more common in pericardial, though we do sometimes have it in endo-cardial troubles.

By these points alone it is difficult and often impossible to differentiate. We must not pay too much attention to the physical signs, to the exclusion of other symptoms, as is too frequently done. If you have pericardial effusion, indicated by an increased area of dullness (the ordinary area being about 2½ inches square), the leaning, of course, is towards pericarditis. So, if you have an area of dullness extending up, say, to the third interspace, you can be sure it is due either to hypertrophy or pericardial effusion. In hypertrophy without dilatation, or true hypertrophy, there will be a strong impulse, while when there is a pericardial effusion the impulse will be weak. When you percuss over a pericardial effusion there will be a *sudden* change from dullness to clearness, while in a dilated heart there will be a *gradual* shading off. If, upon taking a deep inspiration, the dullness disappears, it will incline you to favor a dilated heart, because the forcibly expanded lung will override the heart and give you resonance ; but this will not occur if the dullness be due to pericardial effusion. Pericarditis is rarely idiopathic ; so the history possesses great significance. In rheumatism it may be looked for ; so, also, in the course of Bright's disease, when endo-carditis may also be looked for. In septicæ-

mic diseases, in diphtheria, in typhus fever, small-pox, pyæmia and puerperal fever it may occur, as also in pleurisy as an extension by continuity. Pleurisy and pericarditis may be confounded, but remember that in pleurisy the friction-sound will cease if the breath be held, but not so in pericarditis, and even when, in pleurisy, it is not completely controlled by cessation of respiration, it is much altered and reduced.

We must thus make use of an accumulation of little helps to enable us to draw the line. In pericarditis the friction-sounds are more superficial than in endo-carditis ; in both there is embarrassment in respiration, but it is less marked in acute pericarditis than in acute endo-carditis. In acute endo-carditis the pulse is regular, but frequent, while in pericarditis it is apt to be irregular in consequence of embarrassment to the heart's action ; but such irregularity is also common from emotional causes, so that we should be careful to note whether there are any causes for emotional disturbance.

The treatment of pericarditis is more satisfactory than is that of endo-carditis and consists in the use of good big blisters, that are always satisfactory (4x4) and are more useful than any other procedure. We should also use digitalis to stiffen up the heart, using general supporting treatment and directing special remedies towards the causative disease, whether it be rheumatism or whatever else it is.

